# Blocking C3d^+^/GFAP^+^ A1 Astrocyte Conversion with Semaglutide Attenuates Blood-Brain Barrier Disruption in Mice after Ischemic Stroke

**DOI:** 10.14336/AD.2021.1029

**Published:** 2022-06-01

**Authors:** Qi Zhang, Chang Liu, Rubing Shi, Shiyi Zhou, Huimin Shan, Lidong Deng, Tingting Chen, Yiyan Guo, Zhijun Zhang, Guo-Yuan Yang, Yongting Wang, Yaohui Tang

**Affiliations:** ^1^School of Biomedical Engineering and Shanghai 6^th^ People’s Hospital, Shanghai Jiao Tong University, Shanghai 200030, China.; ^2^Department of Neurology, Ruijin Hospital, School of Medicine, Shanghai Jiao Tong University, Shanghai 200025, China

**Keywords:** A1 astrocyte, blood-brain barrier, ischemia, neuroinflammation, stroke

## Abstract

Astrocytes play an essential role in the modulation of blood-brain barrier function. Neurological diseases induce the transformation of astrocytes into a neurotoxic A1 phenotype, exacerbating brain injury. However, the effect of A1 astrocytes on the BBB dysfunction after stroke is unknown. Adult male ICR mice (n=97) were subjected to 90-minute transient middle cerebral artery occlusion (tMCAO). Immunohistochemical staining of A1 (C3d) and A2 (S100A10) was performed to characterize phenotypic changes in astrocytes over time after tMCAO. The glucagon-like peptide-1 receptor agonist semaglutide was intraperitoneally injected into mice to inhibit A1 astrocytes. Infarct volume, atrophy volume, neurobehavioral outcomes, and BBB permeability were evaluated. RNA-seq was adopted to explore the potential targets and signaling pathways of A1 astrocyte-induced BBB dysfunction. Astrocytic C3d expression was increased, while expression of S100A10 was decreased in the first two weeks after tMCAO, reflecting a shift in the astrocytic phenotype. Semaglutide treatment reduced the expression of CD16/32 in microglia and C3d in astrocytes after ischemic stroke (*p*<0.05). Ischemia-induced brain infarct volume, atrophy volume and neuroinflammation were reduced in the semaglutide-treated mice, and neurobehavioral outcomes were improved compared to control mice (*p*<0.05). We further demonstrated that semaglutide treatment reduced the gap formation of tight junction proteins ZO-1, claudin-5 and occludin, as well as IgG leakage three days following tMCAO (*p*<0.05). *In vitro* experiments revealed that A1 astrocyte-conditioned medium disrupted BBB integrity. RNA-seq showed that A1 astrocytes were enriched in inflammatory factors and chemokines and significantly modulated the TNF and chemokine signaling pathways, which are closely related to barrier damage. We concluded that astrocytes undergo a phenotypic shift over time after ischemic stroke. C3d^+^/GFAP^+^ astrocytes aggravate BBB disruption, suggesting that inhibiting C3d^+^/GFAP^+^ astrocyte formation represents a novel strategy for the treatment of ischemic stroke.

Ischemic stroke is one of the leading causes of morbidity and mortality worldwide. Many pathological processes are involved in stroke progression, including blood-brain barrier (BBB) dysfunction, inflammation [1, 2], excito-toxicity, oxidative stress, neuronal loss, and glial activation. Glial cells, the largest cell population in the central nervous system (CNS) [3], have received extensive attention for their activation in response to CNS injury and subsequent conversion to different phenotypes, including the acquisition of neurotoxic and neuro-protective properties.

Astrocytes play critical roles in maintaining the essential function of the CNS. They instruct the formation and elimination of synapses during development [4, 5], provide trophic factors to support neuronal function [6], mediate the uptake and recycling of neurotransmitters [7], form the structure of the brain and are also involved in the maintenance of BBB integrity [8]. Astrocyte dysfunction is closely related to the pathogenesis of many diseases, including ischemic stroke. Accumulating evidence demonstrates that astrocytes may have different phenotypes. It has been reported that reactive astrocytes can be classified into C3d^+^ A1 and S100A10^+^ A2 phenotypes, which exert neurotoxic and neuroprotective effects, respectively [9, 10]. C3d is a highly upregulated gene in A1 astrocytes, and S100A10 is expressed by ischemic A2 reactive astrocytes [9]. C3d is a fragment of complement component C3 that is induced during complement activation. It plays a critical role in the induction and modulation of immune responses [11]. Various pathogens use C3d to evade the immune system by inhibiting complement activation. By binding to antigens or acting as a protein carrier, C3d increases the in vivo lifespan of antigens, acting as an immune-stimulatory molecule [12]. S100A10 is a unique member of the S100 EF-hand protein family and was first identified within a heterotetrameric complex with annexin A2 [13]. Studies have shown that S100A10 plays an important role in regulating the innate inflammatory response and immune response [14]. S100A10 mediates macrophage recruitment in response to sterile inflammatory stimuli by activating pro matrix metalloproteinase-9, which in turn promotes plasmin-dependent invasion *in vitro* and *in vivo* [15]. A1 astrocytes are induced by interleukin-1 alpha (IL-1α), tumor necrosis factor alpha (TNFα), and the classical complement component C1q, which are secreted by activated microglia. Evidence has shown that C3d^+^ A1 astrocytes lose many normal astrocyte functions, such as promoting neuronal survival, inducing synapse formation/ function, phagocytizing synapses, and releasing toxic factors that kill neurons and oligodendrocytes [16]. However, a comprehensive characterization of astrocyte phenotype conversion and the effects of A1 astrocytes on BBB function after ischemic stroke are still unclear.

The BBB is a highly complex and dynamic structure composed of tight junctions between endothelial cells lining the blood vessels and astrocytic endfeet with a basement membrane, playing a key role in regulating CNS homeostasis [17, 18]. It is well established that the BBB is disrupted after ischemic stroke, which contributes to the development of brain injury and subsequent neurological impairment [19, 20]. Astrocytes directly envelope cerebral microvessels and modulate BBB functions through astrocyte-derived factors and endfeet [21, 22]. Reactive astrocytes secrete not only vascular permeability factors to downregulate tight junction proteins but also factors that protect endothelial cells from apoptosis, suggesting that astrocytes have dual effects on the BBB after CNS injury [23]. Therefore, it is critical to elucidate the role of different astrocyte phenotypes, especially the A1 phenotype, on BBB integrity after ischemic stroke. Novel therapeutic strategies that attenuate BBB dysfunction by reversing A1 astrocytes represent potential treatment options after ischemic stroke.

In this study, we aimed to explore the effect of C3d^+^ A1 astrocytes on BBB integrity after ischemic stroke. We showed for the first time that ischemic stroke increases astrocytic C3d expression, while expression of S100A10 was decreased within 2 weeks after stroke. *In vitro*studies revealed that C3d^+^/GFAP^+^ astrocyte-conditioned medium disrupted BBB integrity, which was potentially mediated by inflammatory factors and chemokines secreted by A1 astrocytes. We also demonstrated that efficiently blocking C3d^+^ A1 astrocyte conversion using semaglutide, a glucagon-like peptide-1 receptor (GLP-1R) agonist, reduces ischemia-induced BBB disruption and improves neurobehavioral recovery. Our study highlights a critical role of C3d^+^ A1 astrocytes in BBB integrity and concludes that C3d^+^ A1 astrocytes represent a potential therapeutic target for treating ischemic stroke.

## MATERIALS AND METHODS

### Animal experiments

**Figure 1.**
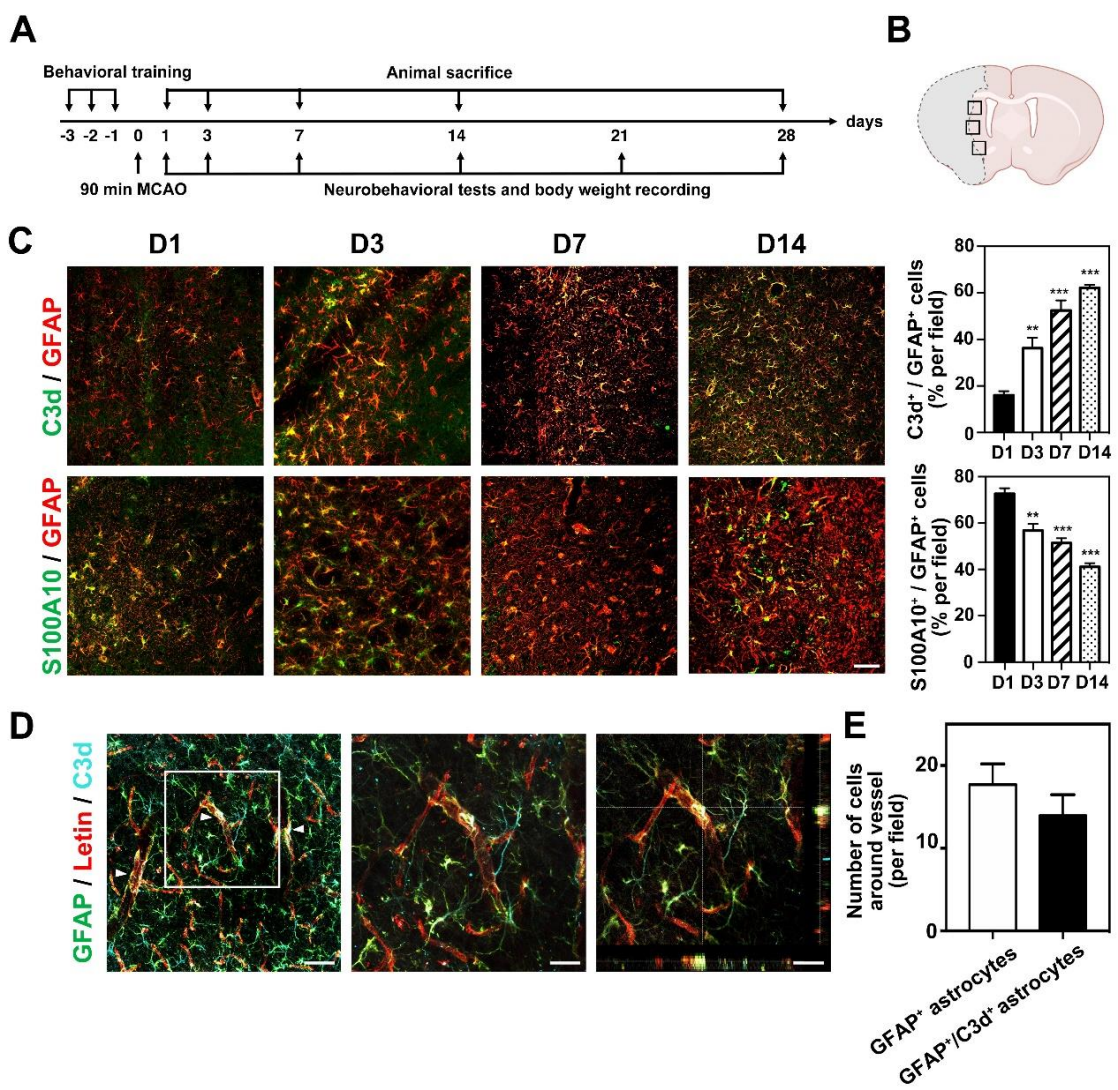
Phenotypic changes of astrocytes after tMCAO in mice. (A) Experimental scheme. Mice were trained for 3 days before tMCAO. Neurobehavioral tests and body weight were examined at 1, 3, 7, 14 and 28 days following tMCAO. Animals were sacrificed at 1, 3, 7, 14 and 28 days following tMCAO. (B) Black boxes showed the peri lesion area for immunostaining and western blot. (C) Photomicrographs showed C3d^+^/GFAP^+^ cells (top, C3d in green color; GFPA in red color) and S100A10^+^/GFAP^+^ cells (bottom, S100A10 in green color; GFAP in red color) in the perifocal area of ipsilateral hemisphere at 1, 3, 7 and 14 days after tMCAO. Bar graph showed the percentage of C3d^+^/GFAP^+^ cells and S100A10^+^/GFAP^+^ in the ipsilateral hemisphere of brain at 1, 3, 7 and 14 days after tMCAO. Scale bar=50 μm. Data are mean±SEM, n=4-5 per group. **p<0.01, ***p<0.001 *vs.* D1. (D) Photomicrographs showed C3d^+^/GFAP^+^ cells closely wrapped around lectin^+^ microvessels (arrowheads) in the perifocal area of the ipsilateral hemisphere in the mouse brain at 3 day after tMCAO. Scale bar=50 μm. (E) Bar graph showed the number of GFAP^+^ astrocytes and C3d^+^/GFAP^+^ astrocytes that co-localized with blood vessels.

Experimental animal studies were performed according to the Animal Research: Reporting of *in vivo* Experiments (ARRIVE) guidelines. Animal experimental protocol was approved by the Institutional Animal Care and Use Committee (IACUC) of Shanghai Jiao Tong University, Shanghai, China. A total of 97 adult male ICR mice weighing 25-30 grams were purchased from Jie Si Jie Laboratory Animal Co., Ltd. (Shanghai, China) and housed in a standard facility with 12-hour light-dark cycle, free access to the food and water, ambient humidity of 20~50%, and temperature of 21~25°C. Mice were randomly assigned to 3 groups, sham group (n=15), transient middle cerebral artery occlusion (tMCAO) group (n=48) and tMCAO with semaglutide treated group (n=34). Experimental designs were summarized in Figure 1A.

### Transient middle cerebral artery occlusion (tMCAO)

The mouse model of tMCAO was established using a method described previously [24]. Briefly, mice were anesthetized with 1.5-2% isoflurane in a mixture of oxygen/nitrous oxide (30%/70%). First, the common carotid artery (CCA), internal carotid artery (ICA), and external carotid artery (ECA) were carefully separated. Then, a 6-0 nylon suture (Dermalon, 1756-31, Covidien) coated with silica gel was inserted through an incision in the ECA, into the ICA, and advanced until the suture tip reached the bifurcation of the middle cerebral artery (MCA). The inserted suture length was about 0.95±0.05 cm. The success of occlusion was confirmed by the decrease of surface cerebral blood flow (CBF) in the MCA territory to 20% of baseline CBF using a laser Doppler flowmetry (Moor Instruments, Devon, UK). Reperfusion was performed by withdrawing the suture 90 minutes after occlusion. The success of reperfusion was confirmed by more than 70% CBF recovered, compared to the baseline. Animals failed in occlusion or reperfusion were excluded from the study. For sham group of ischemic stroke, all the arteries are exposed during the surgical period but the filament is not inserted into the MCA. 5 mice died due to surgical procedures, and 3 animals failed in occlusion or reperfusion were excluded from the study. After surgery, the animals were randomly assigned to the experimental groups.

The animals were randomly assigned to the different groups. The G* Power Analysis was used to estimate and determine the minimal sample size. We calculated the sample size based on other relative studies by using G* Power Priori before the project began. effect size=0.5, α error prob=0.05, power (1-β error prob) =0.8, allocation ratio N1/N2 for t-test =1, the mean value and SD of groups referred from our previous works and other relative studies and calculate the sample size.

### Drug administration

Semaglutide was purchased from China peptides Ltd. Company (Shanghai, China). The amino acid sequence of semaglutide is HXEGTFTSDVSSYLEGQAAKN6-(N-(17-carboxy-1-oxoheptadecyl)-L-gammaglutamyl-2-(2-(2-aminoethoxy) ethoxy) acetyl-2-(2-(2-aminoethoxy) ethoxy) acetyl) EFIAWLVRGRG-OH [25]. 0.2 mg semaglutide was dissolved with 531.2μl PBS and stored as a stock with the concentration of 10^5^ nmol/L. For injection, 7.5μl stock was diluted with 192.5 μl PBS, and then injected into 25g mouse (30nmol/kg) at 2 hours after reperfusion via intraperitoneal (i.p.) injection, followed by the same dose injection every 5 days. The control group received 200 μl of PBS i.p. injection at 2 hours after reperfusion, followed by the same dose injection every 5 days. For in vitro study, 3μl stock was diluted with 10ml medium and 1.5ml medium at the final concentration of 30nmol/L semaglutide was added to each well.

To pharmacological ablate brain microglia, mice were fed PLX5622-formulated AIN-76A diet (1.2?g PLX5622 per kilogram of diet, Plexxikon) for 14 days. Mice were fed normal AIN-76A diet (Plexxikon) as control.

### Neurobehavioral assessment

Neurobehavioral tests were carried out by an investigator blinded to the experimental design using the modified neurological severity score (mNSS), hanging wire test, and rotarod test. mNSS was performed at 1, 3, 7, 14, 21 and 28 days after tMCAO. Hanging wire test and rotarod test were carried out at 3, 7, 14 21 and 28 days after tMCAO. mNSS was a composite score of motor, reflex, and balance tests. The severity score was graded at a scale from 0 to 14, where 0 represents normal and 14 indicates the most severe injury [26].

Hanging wire test was used to assess muscle function and motor coordination [27]. In the hanging test, mice were hung on a horizontal wire. The wire was 1.6?mm in diameter, 50?cm in length, and elevated at 30?cm above the floor. Each mouse was given a score of 10 at the beginning of the test. The mice were then scored based on the number of times they reached the terminal (earn one point) and the number of falls (loss one point) in 180 seconds. If the mouse reached the terminal without falling in one trial, no score was given or taken. Therefore, the highest score an animal could receive is 10. The average score of three 180-second tests was used for analysis. A Kaplan-Meier-like curve was created using the scores, in which 10 indicates best muscle function and motor coordination, while a lower score indicated worse muscle function and motor coordination. Holding impulse (s*g), which was calculated by multiplying body mass (g) and hanging time (s), was used as an outcome measure of overall limb strength.

Rotarod test was used to evaluate motor coordination and balance [28]. Mice were trained for 3 consecutive days before tMCAO. On the first and second day of training, the mice were placed on the non-rotating rod to adapt for 1 minute, then the rod was accelerated to 20 revolutions per minute (rpm) and maintained for 5 minutes. On the third day of training, baseline test after two training sessions was carried out. The rotating rod was accelerated to 40 rpm while the mice were monitored and the fell off rate within 5 minutes was recorded and analyzed.

### Brain infarct volume and atrophy volume measurement

Six mice from each group were euthanized with an excess of 10% chloral hydrate at 3 days and 28 days after tMCAO. Mouse brain samples were collected after intracardial perfusion with 0.1 mol/L PBS followed by 4% paraformaldehyde (PFA, Sinopharm Chemical Reagent, Shanghai, China). Brains were immediately placed in 4% PFA for 6 hours at 4°C and then transferred to 30% sucrose in PBS until the brain sank to the bottom. Processed mouse brains were placed in -42°C pre-chilled isopentane for 10 min and then stored at -80°C. The pre-OCT-embedded brains were cut into floating coronal sections at a thickness of 30 μm from the anterior commissure to the hippocampus and stored in a 24-well plate containing antifreeze (Meilunbio, Dalian, China). Cresyl violet staining was carried out by sampling 16 sections spaced 300 μm apart that collectively spans the entire injury region for each mouse. The selected brain slices were mounted on glass slides and air dried. The sections were then stained in 0.1% Cresyl violet solution (Meilunbio, Dalian, China) followed by de-staining in ethyl alcohol. The ratio of staining in the ipsilateral and contralateral hemispheres was calculated using ImageJ (National Institutes of Health, Bethesda, MD).

Infarct volumes were calculated by the following formula: *V* =∑*h*/3 * [Δ*S_n_* + (Δ*S_n_* * Δ*S_n+1_*)^1/2^ + Δ*S_n+1_*, *V*represents volume. Δ*S* were calculated by subtracting the normal area of the ipsilateral hemisphere from the contralateral hemisphere area. Δ*Sn* and Δ*Sn*+1 represent the infarct areas of two adjacent sections. h represents the thickness between two adjacent brain slices sampled (*h*=300 μm) [29].

### Immunostaining and quantification

Brain slices or cell slides were fixed with 4% PFA for 10 minutes and incubated in 0.3% Triton X-100 solution for 10 minutes followed by blocking with 10% BSA for 1 hour at room temperature. Astrocytes were incubated with antibodies against C3d (1:100, AF2655, R&D system, Minneapolis, MN), S100A10 (1:100, AF2377, R&D system), and glial ﬁbrillary acidic protein (GFAP, 1:200, AB5804, Millipore, MA). Microglia were incubated with antibodies against Iba-1 (1:200, NB100-1028, Novusbio, CO), CD16/32 (1:200, 553141, Thermo fisher scientific, MA), and Arginase (1:200, SC-271430, Santa Cruz Biotechnology, CA). Brain sections were incubated with antibodies against occludin (1:100, 33-1500, Invitrogen, Carlsbad, CA), ZO-1 (1:100, 61-7300, Invitrogen), claudin-5 (1:100, 35-2500, Invitrogen), CD31 (1:200, AF806, R&D Systems) overnight at 4°C. After being washed three times in PBS, brain sections and cell slides were incubated with fluorescent conjugated secondary antibodies for 1 hour at 37°C. The fluorescent images were collected with a TCS SP5 Confocal Scanning System (Leica, Solms, Germany).

Brain sections stained for occludin, ZO-1, or claudin-5 were analyzed for tight junction gap length, which was presented as a percentage (%) of whole tight junction staining. GFAP staining results were analyzed by comparing the GFAP integral optical density (IOD). The number of C3d-positive, S100A10-positive, CD16/32-positive, GFAP-labelled, IBA-1 and CD31 labeled cells was counted from randomly selected fields in the peri-focal striatum of the ipsilateral hemisphere in three mice from each group (Fig. 1B). For each mouse, four sections were evaluated and three fields per section were sampled. The data were analyzed using ImageJ (National Institutes of Health, MD) and Prism Graphpad 8 (San Diego).

IgG leakage was evaluated by performing IgG staining. Brain cryosections were stained with anti-mouse IgG and avidin biotinylated enzyme complex reagent (Vector Labs, Burlingame, CA). 3,3’-diaminobenzidine (DAB) staining was used for the visualisation of IgG signal. Four fields were randomly selected from the area in the peri-focal striatum of the ipsilateral or contralesional hemisphere and analyzed by ImageJ for mean integrated optical density (IOD) analysis.

### Evans blue injection and visualization

At 3 days after tMCAO, EB dye solution (2% EB dye in saline, 4 ml/kg) was slowly injected into the left jugular vein [30]. The mice were sacriﬁced via cardiac perfusion after 2h of EB circulation under anesthetized conditions. Both brain hemispheres were weighed, EB was extracted by incubating plasma or tissue samples with 50% TCA solution (1:3), spinning at 12,000g for 20 min and incubating in ethanol (1:3). The amount of EB was quantiﬁed at 610 nm using a spectrophotometer (Bio-Tek, Winooski, VT).

### Western blotting analysis

The ischemic tissue from the ipsilateral hemisphere of the striatum was dissected and lysed in the protein lysis buffer (RIPA, protease cocktail inhibitor and phosphatase inhibitor) on ice immediately after brain sample collection [31]. The protein concentration of each protein sample was determined by a BCA kit (Meilunbio, Dalian, China). An aliquot of 30 μg of total protein from each sample was loaded onto 12% sodium dodecyl sulfate-polyacrylamide gel electrophoresis (SDS-PAGE) gel and subjected to electrophoresis. The protein samples on the SDS-PAGE gel were transferred to polyvinylidene fluoride membrane (Merck KGaA, Darmstadt, Germany). The membrane was blocked with 5% non-fat milk for 1 hour at room temperature and incubated with the primary antibodies against GFAP (1:4000, AB5804, Millipore, MA), C3d (1:1000, AF2655, R&D system), and β-actin (1:1000, 66009, Invitrogen) overnight at 4°C. After being washed three times in TBST buffer (Meilunbio), the membranes were incubated with HRP-conjugated secondary antibody for 1 hour at room temperature. Immunoblots were developed by incubating with solutions from an enhanced chemiluminescence kit (ECL, Pierce). The results of ECL were analyzed using Image J software.

### Real-Time PCR Analysis

The ischemic tissue extracted from the ipsilateral hemisphere of the striatum was used to isolate total RNA using TRIzol reagent (Invitrogen). The Extracted RNA was used for real-time PCR analysis to detect the expression levels of IL-1α, TNFα, and C1q. Single-strand cDNA was synthesized through a universal cDNA synthesis kit (Qiagen, Hilden, Germany) under the conditions of 42°C for 1 hour and then 95°C for 5 minutes. The expression of RNA was tested by a fast real-time PCR system (7900 HT, ABI, Foster City, CA) using an SYBR Green master mix (Qiagen) with the following cycling conditions: 95°C for 10 minutes followed by 40 cycles of 95°C for 10 seconds and 60°C for 1 minute. GAPDH was used as the control for tissues. The primers used for real-time PCR analysis are listed in Table 1.

**Table 1:** List of primer sequences.

Gene	Forward primer	Reverse primer
IL1α	GCACCTTACACCTACCAGAGT	AAACTTCTGCCTGACGAGCTT
TNFα	ACCCTCACACTCAGATCATCTT	GGTTGTCTTTGAGATCCATGC
C1q	TCTGCACTGTACCCGGCTA	CCCTGGTAAATGTGACCCTTTT
H2-T23	GGACCGCGAATGACATAGC	GCACCTCAGGGTGACTTCAT
Serping1	ACAGCCCCCTCTGAATTCTT	GGATGCTCTCCAAGTTGCTC
H2-D1	TCCGAGATTGTAAAGCGTGAAGA	ACAGGGCAGTGCAGGGATAG
Ligp1	GGGGCAATAGCTCATTGGTA	ACCTCGAAGACATCCCCTTT

### Primary microglia and astrocyte cell cultures

Primary microglia and astrocytes were isolated from postnatal ICR mice (JieSiJie, Shanghai, China). Briefly, the cortex was isolated from the brain of mice and then trypsinized for 10 minutes. After centrifugation, the cell pellet was resuspended in glial cell culture medium with 10% inactivated fetal bovine serum, filtered with a 70 μm filter (Millipore), and then inoculated on the culture dish prepared in advance. The mixed glial cells were kept in an incubator at 37°C and cultured for 10 days under the conditions of 95% humidity and 5% CO_2_. When the astrocytes were confluent, the primary microglia were isolated from the culture by briefly shaking. To obtain primary astrocytes, mixed glial cells were seeded into the petri dish and the medium was replaced with fresh culture medium every 3 days.

Primary astrocytes were cultured under normal culture conditions, and then treated with IL-1α (3?ng/ml, I3901, Sigma, MO), TNF (30?ng/ml, 8902SF, Cell Signaling Technology, MA), C1q (400?ng/ml, MBS143105, MyBio Source, CA) and semaglutide (30 nmol/L, China peptides Ltd. China) for 24 h *in vitro*. Immunostaining of C3d and GFAP was performed and real-time PCR analysis of H2-T23, Serpring1, H2D1 and Ligp1 was conducted to examine whether semaglutide could inhibit IL-1α, TNFα and C1q induced phenotypic change of astrocyte.

### Culture of bEnd.3 cells with different types of astrocyte conditioned medium

Mouse brain capillary endothelial cell line (bEnd.3) cells were cultured under normal culture conditions, and then were cultured with different types of astrocyte-conditional medium (ACM) for 24 hours to prepare for the subsequent experiments. The sources of ACM included medium from the resting A0 astrocytes (A0-CM), from the A1 astrocytes (A1-CM) and from astrocytes treated with medium derived from LPS stimulated microglia (LPS-MCM-AS-CM).

To test if IL-1α (3?ng/ml), TNFα (30?ng/ml) and C1q (400?ng/ml), or LPS have any impact on the tight junction integrity and viability of bEnd.3 cells, we treated bEnd.3 endothelial cells with IL-1α (3?ng/ml), TNFα (30?ng/ml) and C1q (400?ng/ml), or LPS (200 ng/ml, 1μg/ml, 3μg/ml) for 24 hours. Then CCK-8 assay was performed according to the instructions and the expression of tight junction protein ZO-1 was analyzed by immunocytochemistry and western blot.

### RNA sequencing and differentially expressed gene analysis

Total RNA was extracted from cells using Trizol (Invitrogen). RNA purity and quantification were evaluated using the NanoDrop 2000 spectrophotometer (Thermo Scientific). The libraries were constructed using TruSeq Stranded mRNA LT Sample Prep Kit (Illumina, San Diego, CA). The transcriptome sequencing and analysis were conducted by OE Biotech Co., Ltd. (Shanghai, China). The genome-wide transcriptomic analysis was performed on 4 independent experiments in the A0 astrocytes and A1 astrocytes groups. Differential expression analysis was performed using the DESeq (2012) R package. *p*<0.05 and fold change>2 or fold change<0.5 were set as the threshold for significantly differential expression. Gene set enrichment analysis (GSEA; https://www.broadinstitute.org/gsea/index.jsp) was performed to find differential phenotypes between A0 astrocytes and A1 astrocytes groups.

### Statistical analysis

The parametric data were analyzed using Prism Graphpad 8. Comparisons between two groups were carried out using Student's *t*-test. For comparison among multiple groups, statistical significance between each group were examined by one-way ANOVA followed by Bonferroni correction for multiple analyses. All data were expressed as mean±standard error of the mean (SEM), and two-tail *p*<0.05 was considered statistically significant [32].

## RESULTS

### Ischemic stroke induces C3d^+^ expression in astrocytes

To determine whether ischemia initiates a shift in the astrocyte phenotype, we characterized the phenotypic changes of astrocytes at different time points after tMCAO. We found that the number of C3d^+^/GFAP^+^ cells in the ischemic perifocal region gradually increased from day 1 to 14, while the number of S100A10^+^/GFAP^+^ cells gradually decreased (Fig. 1C). The percentage of C3d^+^/GFAP^+^ cells reached≈60% of total cells; in comparison, S100A10^+^/GFAP^+^ cells decreased to≈40% of total cells 14 days after tMCAO, indicating that ischemia shifts the astrocyte phenotype. In addition, we found that on the third day after tMCAO, 17.69±2.50 GFAP^+^ astrocytes and 13.93±2.53 C3d^+^/GFAP^+^ astrocyte endfeet surrounding the microvessels in the perifocal region (Fig. 1D, E), suggesting crosstalk between C3d^+^/GFAP^+^ astrocytes and endothelial cells during tMCAO.

**Figure 2.**
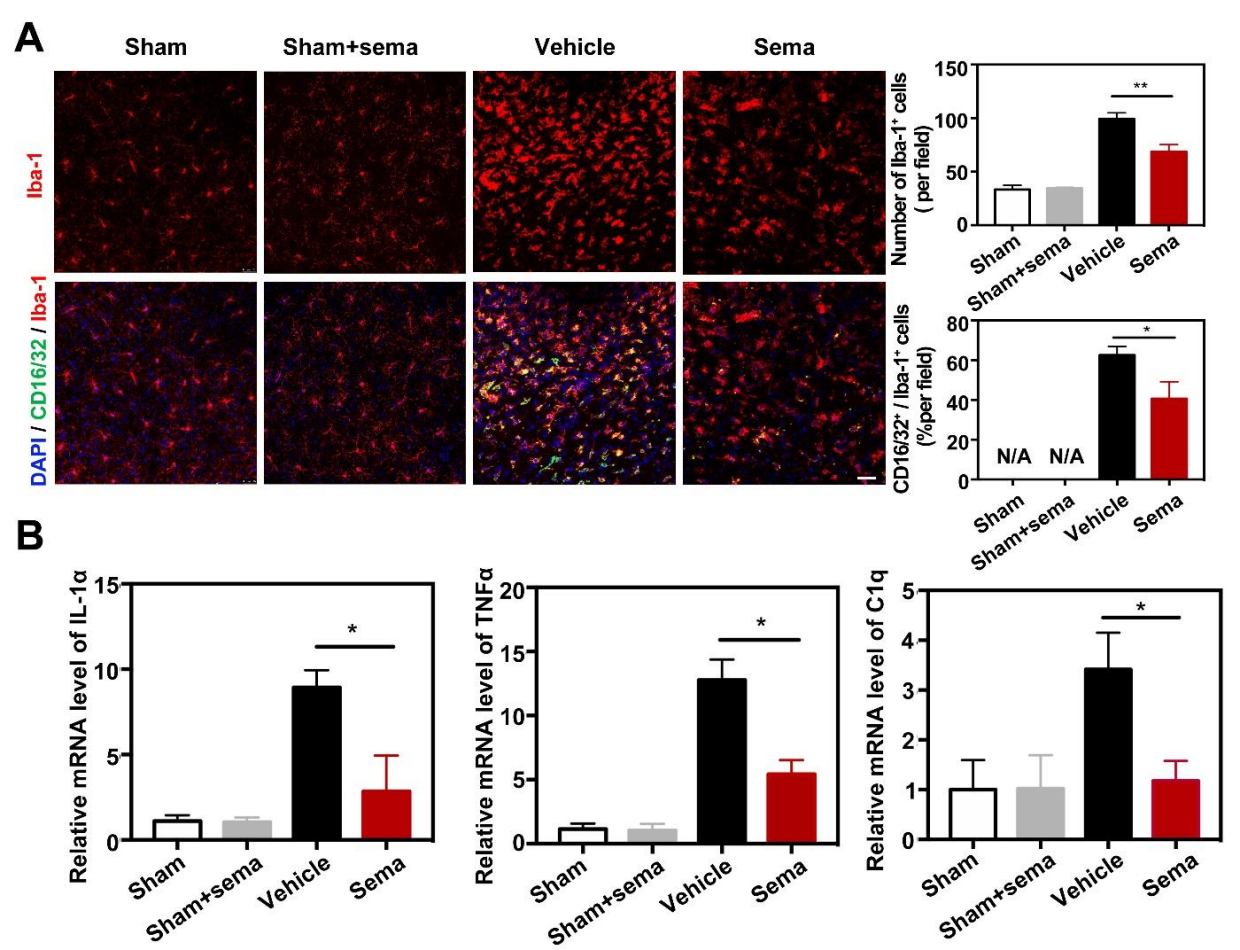
Semaglutide attenuated expression of CD16/32 in microglia after tMCAO. (A) Photomicrographs showed that CD16/32^+^/Iba-1^+^ cells (CD16/32 in green color; Iba-1 In red color) in the ipsilateral hemisphere of the perifocal area in sham mice, sham mice treated with semaglutide, tMCAO mice and semaglutide treated tMCAO mice. Scale bar=25 μm. Bar graphs showed the number of Iba-1^+^ cells and CD16/32^+^/Iba-1^+^ cells in the perifocal area of ipsilateral hemisphere in the semaglutide treated tMCAO mice and control mice. Data are mean±SEM, n=3 per group. *p<0.05, **p<0.01. (B) Bar graphs showed that the mRNA expression of IL-1α, TNFα and C1q in the perifocal area of ipsilateral hemisphere in the semaglutide treated tMCAO mice, the control mice and sham mice treated with semaglutide at day 3 after tMCAO. Data are mean±SEM, n=4 per group, *p<0.05.

### Semaglutide reduces the expression of CD16/32 in microglia and the number of C3d^+^/GFAP^+^ astrocytes after ischemic stroke

GLP1R agonists have been demonstrated to be potential anti-inflammatory agents [33]. Thus, we hypothesized that GLP1R agonists would reduce the number of C3d^+^/GFAP^+^ astrocytes and the expression of CD16/32 in microglia. To test our hypothesis, we injected semaglutide, a GLP1R agonist, into ischemic mice 2 hours after tMCAO. We found that semaglutide treatment significantly reduced the number of Iba-1^+^ cells compared to the control 3 days after tMCAO (*p*<0.01, Fig. 2A). In addition, semaglutide treatment reduced the number of CD16/32^+^/Iba-1^+^ cells compared to the control (*p*<0.05, Fig. 2A), suggesting that semaglutide attenuates microglial M1 polarization during the acute phase after ischemic brain injury.

**Figure 3.**
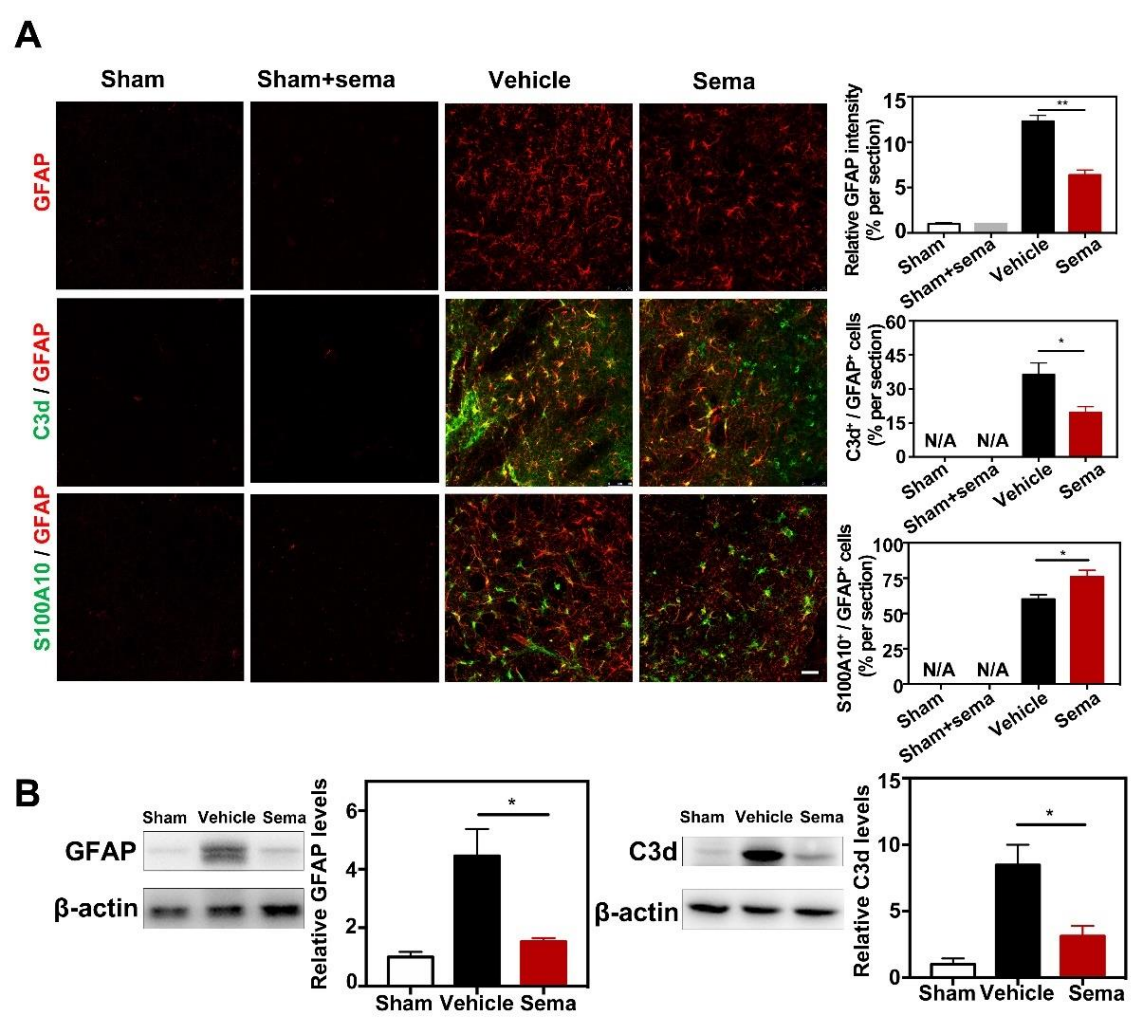
Semaglutide attenuated astrocytic C3d^+^ expression after tMCAO. (A) Representative photomicrographs showed that GFAP^+^ cells, C3d^+^/GFAP^+^ cells (C3d in green color; GFAP in red color) and S100A10^+^/GFAP^+^ cells (S100A10 in green color; GFAP in red color) in perifocal area of ipsilateral hemisphere in the semaglutide treated tMCAO mice, control mice and sham mice treated with semaglutide, at 3 days following tMCAO. Scale bar=50 μm. Bar graph showed that semi-quantification of GFAP intensity, the percentage of C3d+/GFAP+ cells, and S100A10+/GFAP+ cells. Data are presented as mean±SEM, n=3 per group. *p<0.05, **p<0.01. (B) Western blotting analysis data showed relative GFAP and C3d levels in the perifocal area of ipsilateral hemisphere in the semaglutide treated tMCAO mice and control mice. Data are mean±SEM, n=3 per group. *p<0.05.

It has been reported that IL-1α, TNFα and C1q together trigger A1 conversion of reactive astrocytes and subsequently cause neuronal death. Therefore, we examined expression of IL-1α, TNFα and C1q after tMCAO. Real-time PCR analysis of the perifocal region of the ipsilateral hemisphere revealed that tMCAO induced IL-1α, TNFα and C1q hyperexpression (Fig. 2B), and expression of these factors was reduced in the semaglutide-treated mice compared to controls (*p*<0.05, Fig. 2B). To validate whether semaglutide reduces the expression of C3d in astrocytes, western blotting and immunofluorescence staining were performed to detect C3d, S100A10 and GFAP protein levels. We found that astrocytic C3d and GFAP expression apparently increased after ischemic brain injury (*p*<0.001, Fig. 3A, B), and semaglutide treatment decreased their expression 3 days after tMCAO (*p*<0.01, Fig. 3A, B). In contrast, expression of S100A10 increased significantly in the semaglutide treatment group 3 days after injury (*p*<0.05, Fig. 3A, B).

To validate whether the formation of C3d^+^/GFAP^+^ reactive astrocytes is induced by activated microglia in vivo, we generated microglial depletion models of the mouse brain [34]. We fed mice in different groups a PLX5622-formulated diet for 14 days. After continuous microglial depletion for 14 days, only a few microglia were found in the brain (Supplementary Fig. 2A). Then, we observed that microglial-depleted mice failed to produce C3d^+^/GFAP^+^ astrocytes after tMCAO (Supplementary Fig. 2B), indicating that reactive microglia induce C3d^+^/GFAP^+^ astrocytes.

**Figure 4.**
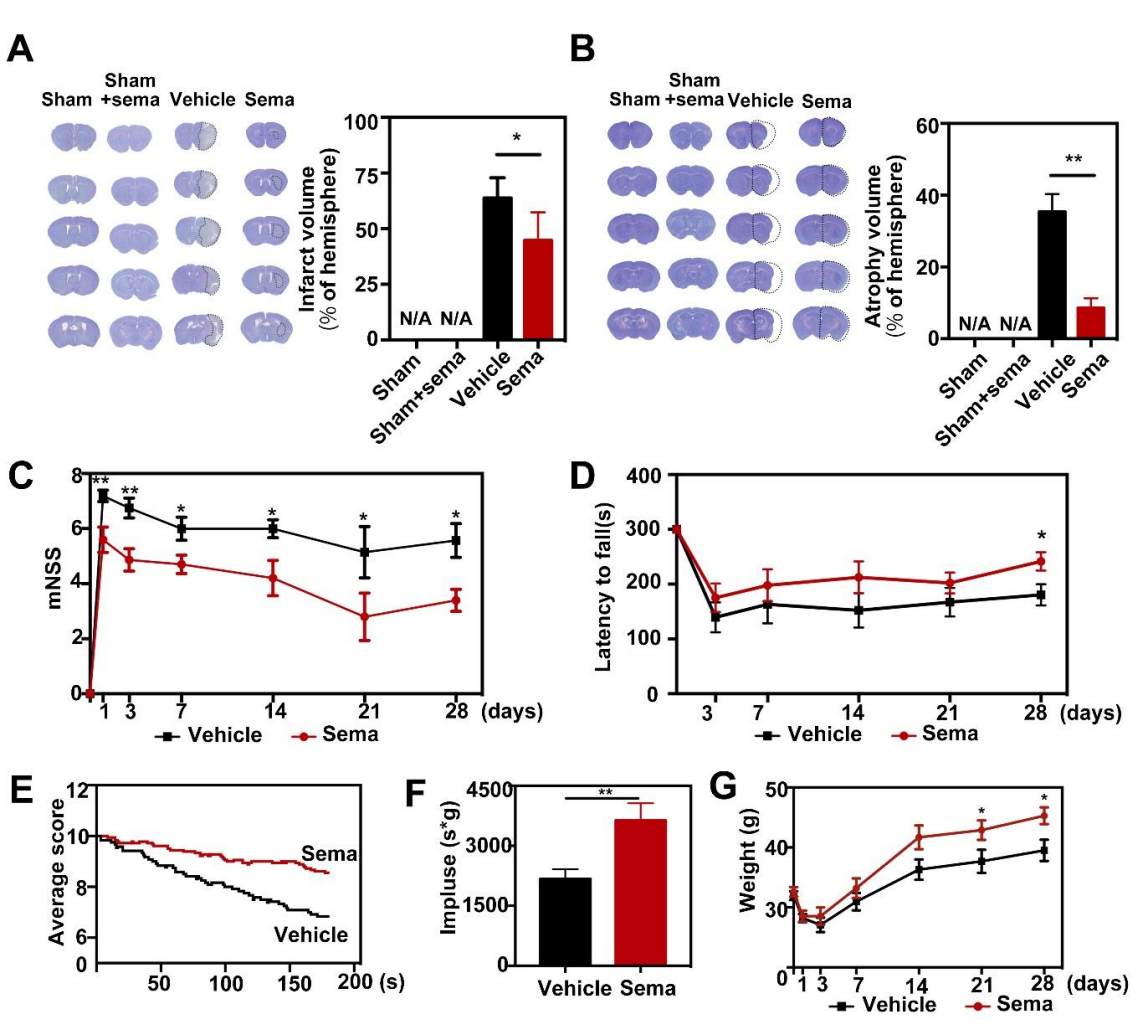
Inhibiting C3d^+^/GFAP^+^ astrocytes formation attenuated infarct volume and neurobehavioral deficit in the semaglutide treated tMCAO mice. (A-B) Cresyl violet-stained coronal sections of the brain in control mice, sham mice treated with semaglutide, tMCAO mice, and semaglutide treated tMCAO mice following 3 (A, infarct) and 28 days (B, atrophy) of tMCAO. The brain infarct area and brain atrophy were circled by the dashed line. Bar graph showed the semi-quantitative analysis of the infarct volume and atrophy volume. Data are mean±SEM, n=6 per group. *p<0.05, **p<0.01. (C-F) Neurobehavioral outcomes were assessed by three neurobehavioral tests including the modified neurological severity score (mNSS, C), rotarod test (D), and hanging wire test (E-F). Line graph showed body weight (G), Data are mean±SEM, n=9-12 per group, **p<0.01, *p<0.05.

### Inhibiting C3d^+^/GFAP^+^ A1 astrocyte formation reduces brain infarct volume and improves neurobehavioral outcomes after tMCAO in mice

To determine the effect of blocking C3d^+^/GFAP^+^ A1 astrocyte formation on infarction and neurobehavioral recovery in stroke mice, we examined infarct volume and neurobehavioral parameters, including the mNSS score, rotarod test and hanging wire test, in tMCAO mice. We found that infarct volume was greatly decreased in semaglutide-treated mice compared to controls 3 days after tMCAO (*p*<0.05, Fig. 4A). Similarly, atrophy volume was reduced in semaglutide-treated mice compared to controls 28 days after tMCAO (*p*<0.01, Fig. 4B). Furthermore, we found that the mNSS scores were lower in semaglutide-treated mice than in the controls 1, 3, 7, 14, 21 and 28 days following tMCAO (*p*<0.05, Fig. 4C). Furthermore, motor functions as assessed by the rotarod test and hanging wire test were better in the semaglutide-treated mice than in control mice (*p*<0.05, Fig. 4D, F). Changes in body weight in semaglutide-treated mice were significantly recovered 21 and 28 days after tMCAO (*p*<0.05, Fig. 4G), suggesting that general condition was also improved in semaglutide-treated mice after tMCAO.

**Figure 5.**
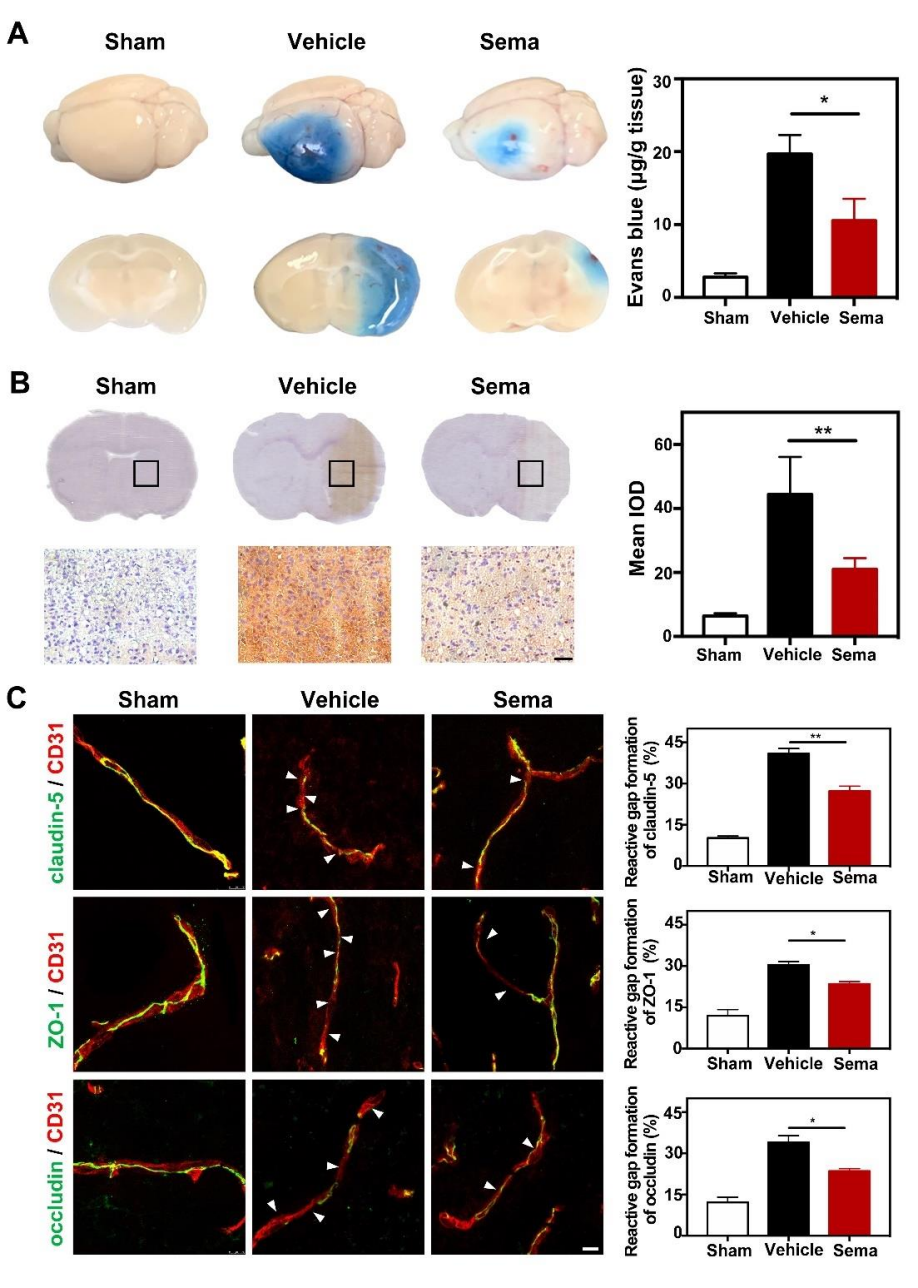
Inhibiting C3d^+^/GFAP^+^ astrocytes formation reduced BBB disruption and gap formation of tight junction in tMCAO mice. (A) Photographs shows Evans blue (EB) exudation for the whole brain and brain sections in the semaglutide treated tMCAO mice, tMCAO mice, and control mice at 3 days after tMCAO. The value of extravasated EB was recorded by a spectrophotometer at 610 nm. Bar graph showed the quantitative analysis of Evans blue leakage in these 3 groups. Data are mean±SEM, n=3-5 per group. *p<0.05. (B) Photographs showed IgG staining in the coronal section of the brain following 3 days of tMCAO in the semaglutide treated tMCAO mice, tMCAO mice, and control mice. Scale bar=50 μm. Bar graph showed the mean IOD of IgG intensity in these 3 groups. Data are mean±SEM, n=6 per group. **p<0.01. (C) Photomicrographs showed CD31/claudin-5, CD31/ZO-1, and CD31/occludin double staining in the ischemic peri-focal areas in the semaglutide treated tMCAO mice, tMCAO mice, and control mice. White arrows indicated discontinuous labeling and gap formation. Scale bar=10 μm. Bar graph showed relative gap formation of ZO-1, claudin-5, and occludin. Data are mean±SEM, n=3 per group. *p<0.05, **p<0.01

**Figure 6.**
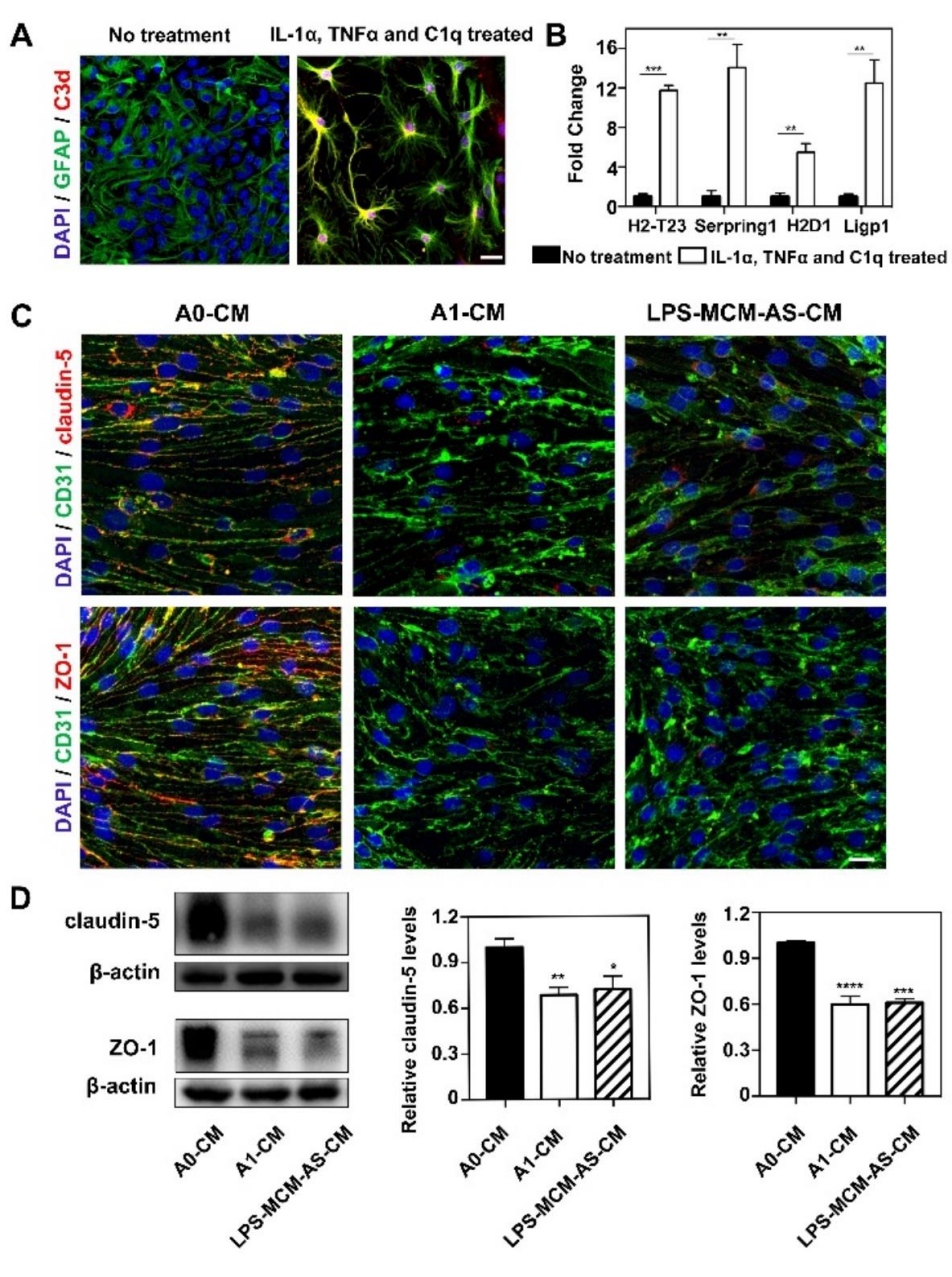
C3d^+^/GFAP^+^ astrocyte derived medium reduced tight junction protein expression *in vitro.* (A) Immunofluorescence images showed resting astrocytes were converted to C3d^+^/GFAP*^+^* cells (C3d in red color; GFAP in green color; DAPI in blue color) after treated with IL-1α, TNFα and C1q. Scale bar=25 μm. (B) Bar graph showed the mRNA levels of C3d^+^/GFAP*^+^* cells related genes H2-T23, Serping1, H2D1 and Ligp1 expression after IL-1α, TNFα and C1q treatment. Data are mean±SEM. n=3 per group. *p<0.05, ***p<0.001. (C) Photomicrographs showed tight junction proteins (claudin-5 and ZO-1 in red color) expressed in CD31*^+^* cells (green) that were treated by 1) medium derived from inactivated astrocytes (A0-CM), 2) medium derived from IL-1α, TNFα and C1q treated astrocytes (A1-CM) and 3) medium derived from astrocytes that were treated with LPS-stimulated microglia (LPS-MCM-AS-CM). Scale bar=25 μm. (D) Western blotting analysis of claudin-5 and ZO-1 in the A0-CM group, A1-CM group and LPS-MCM-AS-CM group. n=3 per group. *p<0.05, **p<0.01, ***p<0.001, ****p<0.0001.

### Inhibiting C3d^+^/GFAP^+^ A1 astrocyte formation attenuates BBB disruption after tMCAO in mice

To evaluate the effect of C3d^+^/GFAP^+^ A1 astrocytes on the BBB after tMCAO, we examined Evans blue (EB) extravasation in the mouse brain 3 days after tMCAO. EB dye is always used as a marker of albumin effluxion to evaluate BBB permeability, which can easily permeate the BBB after brain injury. We found that EB extravasation was limited to the striatum of the ipsilateral hemisphere after semaglutide treatment, while EB extravasation was detected in both the striatum and cortex in the control group (Fig. 5A). Quantiﬁcation of EB results showed that semaglutide drastically reduced the total amount of extravasated EB compared to that in the control group 3 days after tMCAO (*p*<0.01, Fig. 5A). To further evaluate BBB permeability, we examined IgG leakage in the mouse brain 3 days after tMCAO. The results showed that IgG leakage was reduced in the ipsilateral hemisphere in semaglutide-treated mice compared to controls (*p*<0.01, Fig. 5B). Immunostaining of tight junction proteins, including occludin, ZO-1 and claudin-5, in combination with the endothelial cell marker CD31 was used to assess endothelial tight junction gap formation. We observed disruption of junction arrangement and microvessel gap formation in the perifocal region of the tMCAO mouse brain. However, the gap formation distance was decreased in semaglutide-treated mice compared to control mice (*p*<0.05, Fig. 5C), suggesting a reduction in tight junction gap formation in the perifocal region during the acute phase after tMCAO.

### C3d^+^/GFAP^+^ A1 astrocyte-derived medium reduces tight junction protein expression in endothelial cells in vitro

It was previously reported that IL-1α, TNFα and C1q are required for converting resting A0 astrocytes into A1 astrocytes [10]. Our immunofluorescence staining images and real-time PCR results showed that IL-1α, TNFα and C1q treatment increased the expression of C3d and related genes in astrocytes, including H2-T23, Serping1, H2D1 and Ligp1 (*p*<0.01, Fig. 6A, B), suggesting that we successfully induced A1 astrocytes *in vitro*.

To investigate the effect of A1 astrocytes on the tight junction proteins of endothelial cells, bEnd.3 cells were treated with medium from normal astrocytes (A0-CM), medium from IL-1α-, TNFα- and C1q-treated astrocytes (A1-CM) or medium from astrocytes treated with LPS-stimulated microglia (LPS-MCM-AS-CM). We found that A1-CM treatment and LPS-MCM-AS-CM treatment reduced ZO-1 and claudin-5 expression in endothelial cells compared to controls (A0-CM treatment) (Fig. 6C, D). In addition, as shown in Supplementary Fig. 4, expression of IL-1alpha, TNF-alpha and C1q was increased in primary murine microglia after LPS treatment. Furthermore, adding LPS-treated microglial medium to astrocytes significantly increased C3d expression and A1 astrocyte-related gene expression (Supplementary Fig. 1D-F), suggesting that LPS-treated microglial medium is sufficient to trigger A0-to-A1 conversion *in vitro*.

In our study, astrocytes were exposed to IL-1α, TNFα and C1q for 24 hours, and the medium was collected without medium exchange and added to bEnd.3 endothelial cells (Supplementary Fig. 3A). To exclude the A1 cocktail or LPS alone having any significant impact on the TJ integrity and viability of the bEnd.3 monolayer, we treated bEnd.3 endothelial cells with IL-1α (3?ng/ml), TNFα (30?ng/ml) and C1q (400?ng/ml), or LPS (200 ng/ml). Our results showed that addition of the A1 cocktail or LPS did not affect the expression of ZO-1 (Supplementary Fig. 3B, C) or the viability of bEnd.3 endothelial cells (Supplementary Fig. 3D).

To determine whether semaglutide treatment attenuates the loss of TJ integrity and the viability of bEnd.3 cells exposed to LPS independent of the blockage of A1 astrocyte polarization, bEnd.3 cells were first exposed to different concentrations of LPS (200 ng/ml, 1 μg/ml and 3 μg/ml). Our CCK-8 assay revealed that 200 ng/ml and 1 μg/ml LPS did not have any effect on the viability of bEnd.3 cells (Supplementary Fig. 3D, Supplementary Fig. 4B), but 3 μg/ml LPS treatment significantly reduced cell viability (Supplementary Fig. 4B). In addition, semaglutide treatment slightly increased the viability of bEnd.3 cells in response to 3 μg/ml LPS stimulation (Supplementary Fig. 4B). Our western blot data showed that 3 μg/ml LPS treatment decreased expression of the TJ protein ZO-1, while semaglutide treatment increased the expression of ZO-1 (Supplementary Fig. 4C).

### Semaglutide treatment does not directly prevent IL-1α-, TNFα- or C1q-induced phenotypic changes in astrocytes

To determine whether semaglutide directly blocks the phenotypic change of astrocytes, we treated astrocytes with IL-1α+TNFα+C1q or with IL-1α+TNFα+C1q+ semaglutide (Supplementary Fig. 1A). Our *in vitro* data showed that IL-1α, TNFα and C1q treatment increased the number of C3d^+^/GFAP^+^ astrocytes (Supplementary Fig. 1B) and increased the expression of H2-T23, Serpring1, H2D1 and Ligp1 (Supplementary Fig. 1C). However, the addition of semaglutide did not affect the number of C3d^+^/GFAP^+^ astrocytes or the expression of those genes (Supplementary Fig. 1B, C). Interestingly, when we treated astrocytes with medium derived from LPS-stimulated microglia or LPS+semaglutide-stimulated microglia (Supplementary Fig. 1D), we found that semaglutide reduced the number of C3d^+^/GFAP^+^ astrocytes (Supplementary Fig. 1E) and decreased the expression of H2-T23, Serpring1, H2D1 and Ligp1 (Supplementary Fig. 1F). Our data suggested that semaglutide blocks the phenotypic shift of astrocytes by inhibiting the activation of microglia but does not directly affect astrocyte phenotype.

### IL-1α, TNFα and C1q treatment upregulate inflammatory-, immune- and chemotaxis-related signaling pathways in astrocytes

**Figure 7.**
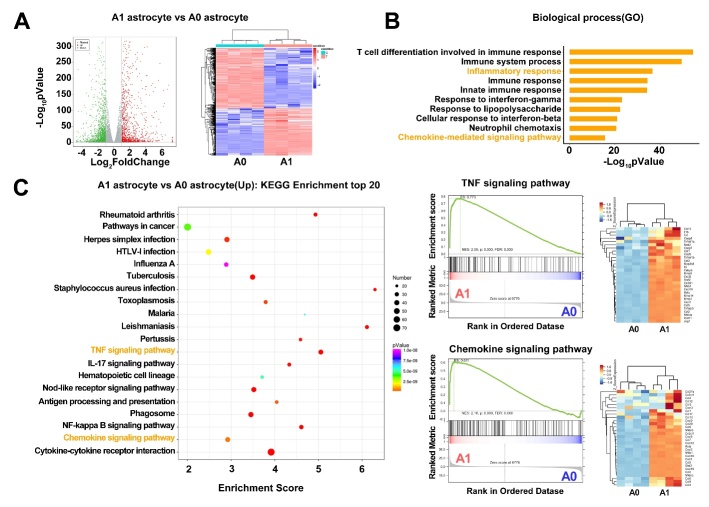
IL-1α, TNFα and C1q treated astrocyte expressed different transcripts in RNA-seq data. (A) Volcano plot showed the upregulated (red) and downregulated (green) genes between A1 and A0 astrocyte. The horizontal axis is log_2_fold change, and the vertical axis is -log_10_*p* value, *p*<0.05. Heatmap showed the overall distribution of differentially expressed genes. Gene expression data was colored red for high expression and blue for low expression. (B) GO enrichment analysis of differentially expressed genes based on RNA-seq data with p-value showed top 10 upregulated biological process. (C) KEGG enrichment top 20 analysis suggested that TNF signaling pathway and cytokine-cytokine receptor interaction were related with the phenotypic changes of astrocytes.

To explore the underlying molecular mechanisms of how A1 astrocytes disrupt BBB integrity, we analyzed transcriptome differences between IL-1α-, TNFα- and C1q-treated astrocytes and nontreated astrocytes. We found that 2474 genes exhibited differentially expression (fold change>2) in IL-1α-, TNFα- and C1q-treated astrocytes, among which 1085 genes were upregulated, and 1389 genes were downregulated (Fig. 7A). Among them, BBB breakdown-related genes, including matrix metalloproteinase-3 (MMP-3) and MMP-9, were highly upregulated.

Gene Ontology (GO) pathway enrichment analyses of the upregulated genes showed that compared to nontreated astrocytes, multiple altered biological processes, including T cell differentiation, immune response, inflammatory response and chemokine-mediated signaling pathway, were increased in IL-1α-, TNFα- and C1q-treated astrocytes (Fig. 7B). KEGG analyses further demonstrated that canonical signaling pathways related to inflammation, chemokine signaling, and the immune response were enriched in IL-1α-, TNFα- and C1q-treated astrocytes (Fig. 7C). Many inflammatory factors and chemokines were upregulated in IL-1α-, TNFα- and C1q-treated astrocytes, indicating that A1 astrocytes may induce inflammatory and immune responses to disrupt BBB integrity after stroke.

## DISCUSSION

In the present study, we demonstrated that the number of C3d^+^/GFAP^+^ A1 astrocytes increased within 14 days after tMCAO, while S100A10^+^/GFAP^+^ A2 astrocytes decreased during this time. Semaglutide treatment reduced the number of CD16/32^+^ microglia and C3d^+^/GFAP^+^ A1 astrocytes and subsequently reduced brain infarct volume, improved neurobehavioral outcomes, reduced neuroinflammation, and attenuated BBB disruption in tMCAO mice. Further RNA-seq experiments revealed that inflammatory-, immune- and chemotaxis-related signaling pathways are involved in A1 astrocyte function. Our results suggested that C3d^+^/GFAP^+^ A1 astrocytes represent a specific group that plays an important role in BBB integrity. Inhibiting astrocyte conversion provides a novel approach for ischemic stroke therapy.

Astrocytes play essential roles in the formation and maintenance of the BBB [35-37]. Accumulating evidence has demonstrated that astrocytes are heterogeneous in the CNS, both morphologically and functionally [38-40]. In particular, the recently proposed disease-associated astrocytes have attracted widespread attention [13]. IL-1α, TNFα, and C1q can activate astrocytes to transform into a neurotoxic A1 phenotype [10]. However, the effect of this phenotypic conversion on BBB integrity has been largely unexplored. Using a tMCAO model, we demonstrated that resting A0 astrocytes transformed into the A1 phenotype after tMCAO, and the number of A1 astrocytes increased over time, while inhibition of A1 astrocyte transformation by semaglutide attenuated BBB disruption. The results of *in vitro* experiments showed that conditioned medium from A1 astrocytes reduced tight junction protein expression in endothelial cells, suggesting the destructive effect of A1 astrocytes on BBB integrity. However, in our study, the effect of semaglutide on neuroprotection was not explored, and whether semaglutide attenuates BBB damage by facilitating its neuroprotective effects needs further investigation.

IL-1α, TNFα and C1q are three inflammatory factors that are specifically expressed in microglia that are required for A1 astrocyte transformation [16]. Our results demonstrated that IL-1α, TNFα, and C1q were highly upregulated in the brain 3 days after tMCAO and increased of A1 astrocyte transformation was detected in the perifocal area after ischemic stroke, while this phenomenon was absent in the nonstroke brain, indicating that ischemic stroke-activated microglia could release these cytokines and promote resting A0 astrocytes toward an A1 astrocyte transformation. Our *in vitro*study further showed that addition of IL-1α, TNFα and C1q into the medium of resting astrocytes promotes A1 astrocyte transformation.

Astrocytes play important roles in maintaining BBB integrity by safeguarding endothelial tight junctions [41-43]. We found that 3 days after ischemic stroke, a large number of A1 astrocyte endfeet was wrapped around the wall of blood vessels, leading us to suspect potential crosstalk between A1 astrocytes and endothelial cells, which may affect BBB integrity. Our data strongly supported this hypothesis, as conditioned medium derived from A1 astrocytes or LPS-MCM-treated astrocytes reduced tight junction proteins in endothelial cells, suggesting that toxic factors secreted from A1 astrocytes degrade tight junction proteins.

To gain insight into the A1 astrocyte-derived toxic factors that disrupt BBB integrity, transcriptomic analyses of A0 and A1 astrocytes were performed. Our data suggested that multiple biological processes related to BBB function were increased in A1 astrocytes. Growing evidence has shown that inflammatory and immune responses are two key driving forces that critically affect BBB function after stroke. Inflammation is involved in tight junction decomposition, which ultimately causes BBB breakdown. We demonstrated that the TNF, IL-17 and NF-kappa B signaling pathways were enriched in A1 astrocytes, and many inflammatory factors, such as TNF and IL-6, were increased. It should be noted that matrix metalloproteinases (MMPs), including MMP9, MMP14 and MMP3, were highly upregulated in A1 astrocytes as well. MMPs are zinc-dependent endopeptidases produced by astrocytes, microglia, and endothelial cells that degrade extracellular matrix proteins during ischemic stroke [44]. MMP9 and MMP3 are reported to exert detrimental effects on BBB integrity. Blocking MMP9 and MMP3 significantly attenuates BBB disruption and improves neurobehavioral recovery [45]. A1 astrocytes may induce BBB disruption by MMP9 and MMP3, as we found that MMP9 and MMP3 were upregulated in A1 astrocytes.

In addition to the inflammatory response, chemokine signaling pathways and chemokines, including CCL12, CCL5, CXCL1, CXCL2 and CXCL10, were also enriched in A1 astrocytes. These chemokines mediate inflammatory and immune responses by guiding immune cell migration into the lesion area of the brain. For example, CCL5 is a chemoattractant of T cells to the site of inflammation, which mediates cerebral inflammation and causes BBB disruption [46]. CXCL1 produced by astrocytes is a critical ligand required for neutrophil transendothelial migration and exacerbates brain damage[47]. Activating MMPs degrade endothelial cell tight junction proteins, secrete inflammatory cytokines and adhesion molecules, and induce endothelial cell death [44]. These processes are all closely related to BBB disruption.

In addition to A1 astrocytes, A2 astrocytes are also detected in the brain after ischemic stroke. Previous studies have shown that activated astrocytes promote neuronal function recovery and repair [48-50]. A2 astrocytes upregulate neurotrophic factors and vascular growth factors, which presumably promote neuronal survival, aid axon regeneration, and promote BBB repair [51]. Therefore, manipulating the astrocyte phenotype is regarded as a potential therapeutic strategy. Understanding the heterogeneity of astrocytes helps to comprehend how the function of astrocytes shapes the function and dysfunction of the brain.

GLP-1 is a 30-amino acid peptide hormone that stimulates insulin secretion. Currently, GLP-1 and GLP-1 receptor agonists, including exendin-4, liraglutide, and semaglutide, have been developed and are approved for treating type 2 diabetes [52, 53]. In addition, recent studies have demonstrated their potential for neurological disorder treatment [54]. In a recent study, semaglutide was shown to have neuroprotective effects in a rat MCAO model [25]. In addition, a previous study demonstrated that NLY01, a GLP1R agonist, protects dopaminergic neurons from death and improves behavioral recovery in Parkinson’s disease, which is mediated by the block of A1 astrocyte transformation [10]. In the present study, we found that similar to NLY01, semaglutide also reduces A1 astrocyte transformation. This may be caused by attenuation of microglial inflammation, as semaglutide treatment reduced the expression of IL-1α, TNFα, and C1q and M1 microglial polarization. Together, these results highlight the important role of A1 astrocytes in BBB disruption after ischemic stroke.

## Conclusions

Our study demonstrated that stroke-induced C3d^+^/GFAP^+^ A1 astrocytes disrupt blood-brain barrier integrity and that blocking C3d^+^/GFAP^+^ A1 astrocyte formation using semaglutide attenuates brain injury, suggesting the detrimental role of C3d^+^/GFAP^+^ A1 astrocytes in stroke. We envision that C3d^+^/GFAP^+^ A1 astrocytes represent a novel therapeutic target for ischemic stroke therapy.

## Ethics approval

Animal protocol was approved by the Institutional Animal Care and Use Committee (IACUC) of Shanghai Jiao Tong University, Shanghai, China. All animal procedures were performed to minimize pain or discomfort in accordance with current protocols.

## Availability of data and materials

The datasets used and/or analyzed during the current study are available from the corresponding author on reasonable request.

## Supplementary Materials

The Supplementary data can be found online at: www.aginganddisease.org/EN/10.14336/AD.2021.1029.


