# Supplementary material for: Blocking C3d+/GFAP+ A1 Astrocyte Conversion with Semaglutide Attenuates Blood-Brain Barrier Disruption in Mice after Ischemic Stroke
Source: Aging Dis. 2022 Jun 1;13(3):943–59. doi: 10.14336/AD.2021.1029 (PMC9116904; doi:10.14336/AD.2021.1029)
Supplement: Supplementary file 1 [file AD-13-3-943-s.pdf]

SUPPLEMENTARY DATA

Blocking C3d+/GFAP+ A1 Astrocyte Conversion with Semaglutide Attenuates Blood-Brain Barrier Disruption in Mice after Ischemic Stroke

Qi Zhang[1,#], Chang Liu[1,#], Rubing Shi[1], Shiyi Zhou[1], Huimin Shan[1], Lidong Deng[1], Tingting Chen[1], Yiyan Guo[1], Zhijun Zhang[1], Guo-Yuan Yang[1,2], Yongting Wang[1*], Yaohui Tang[1*]

© 2021. Zhang Q et al. Published online at http://www.aginganddisease.org/EN/10.14336/AD.2021.1029

SUPPLEMENTARY DATA

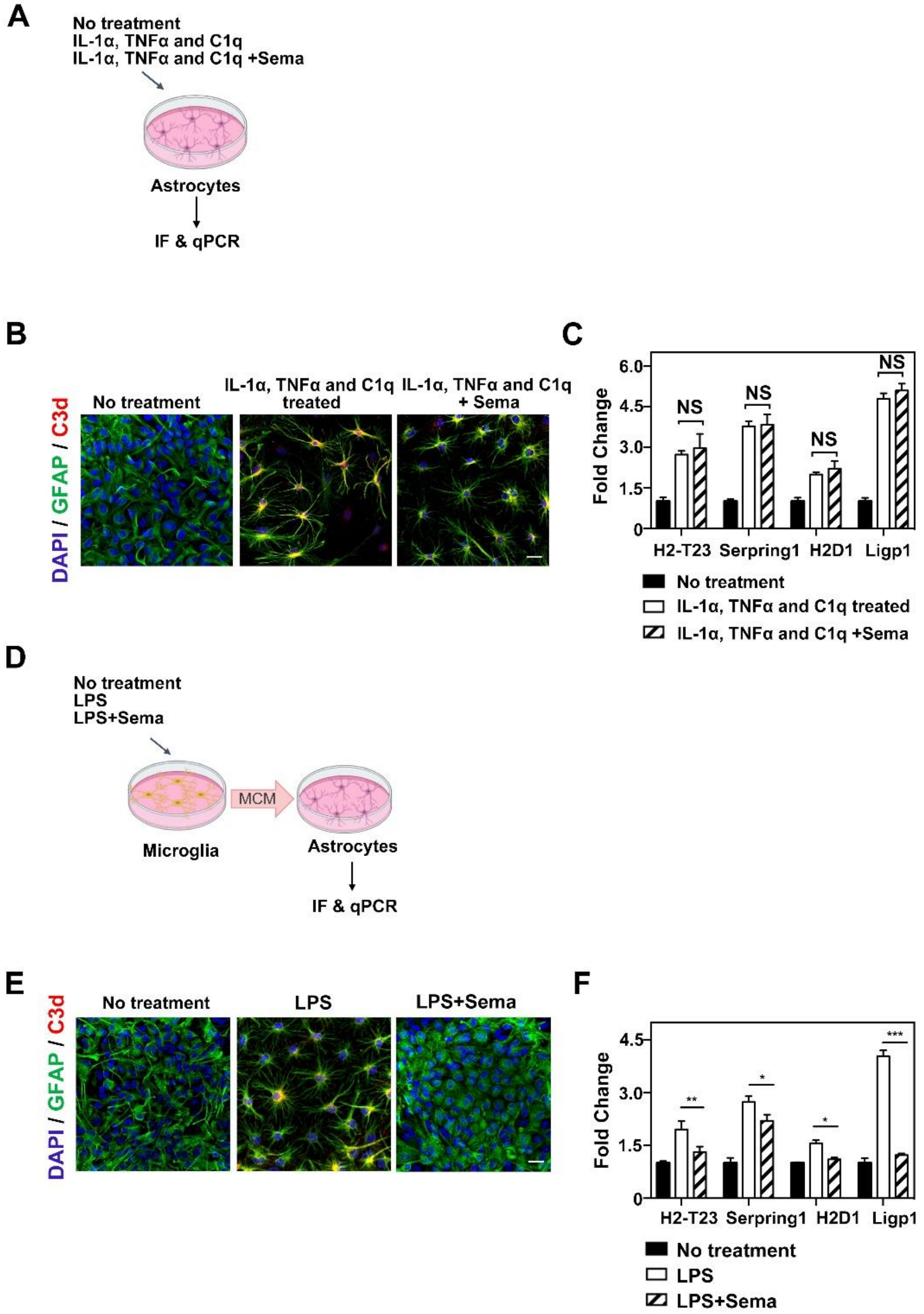

Supplementary **Figure 1. Semaglutide treatment did not directly prevent IL-1α, TNFα and C1q induced phenotype change of astrocyte. A.** Astrocytes were treated with IL-1α+TNFα+C1q or IL-1α+TNFα+C1q+semaglutide for 24 hours and immunocytochemistry and real-time PCR were performed. **B.** Immunostaining of C3d$^+$/GFAP$^+$ cells (C3d in red color; GFAP in green color; DAPI in blue color). Scale bar=25 µm. **C.** Bar graph showed the mRNA levels of C3d$^+$/GFAP$^+$ cells related genes H2-T23, Serping1, H2D1 and Ligp1 expression after IL-1α+TNFα+C1q or IL-1α+TNFα+C1q+semaglutide treatment. Data are mean ± SEM. n=3 per group. NS, p>0.05. **D.** Astrocytes were treated with the medium derived from LPS stimulated microglia or LPS+semaglutide stimulated microglia for 24 hours and the immunocytochemistry and real-time PCR were performed. **E.** Immunostaining of C3d$^+$/GFAP$^+$ cells (C3d in red color; GFAP in green color; DAPI in blue color). Scale bar=25 µm. **F.** Bar graph showed the mRNA levels of C3d$^+$/GFAP$^+$ cells related genes H2-T23, Serping1, H2D1 and Ligp1 expression after treatment with the medium derived from LPS stimulated microglia or LPS+semaglutide stimulated microglia. Data are mean±SEM. n=3 per group. *, *p*<0.05; **, *p*<0.01; ***, *p*<0.001.

© 2021. Zhang Q et al. Published online at http://www.aginganddisease.org/EN/10.14336/AD.2021.1029

SUPPLEMENTARY DATA

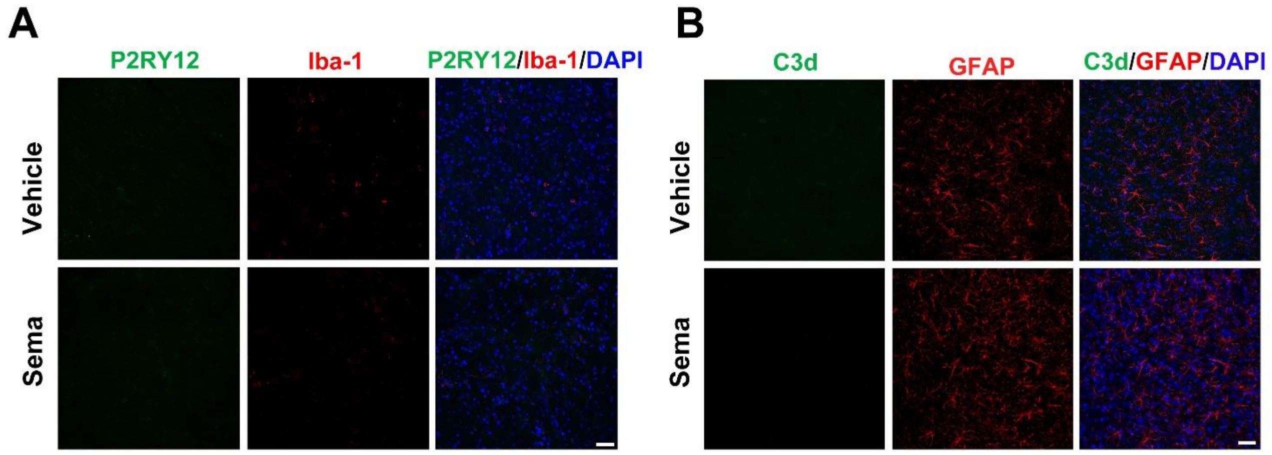

Supplementary Figure 2. The formation of C3d+/GFAP+ reactive astrocytes are induced by activated microglia. A. Photomicrographs showed that P2RY12^{+}/Iba-1^{+} cells (P2RY12 in green color; Iba-1 In red color; DAPI in blue color) in the ipsilateral hemisphere of the perifocal area in tMCAO mice and semaglutide treated tMCAO mice. Scale bar=75 μm. **B.** Immunofluorescence images showed C3d^{+}/GFAP^{+} cells (C3d in red color; GFAP in green color; DAPI in blue color) after microglial depletion. Scale bar=75 μm.

© 2021. Zhang Q et al. Published online at http://www.aginganddisease.org/EN/10.14336/AD.2021.1029

SUPPLEMENTARY DATA

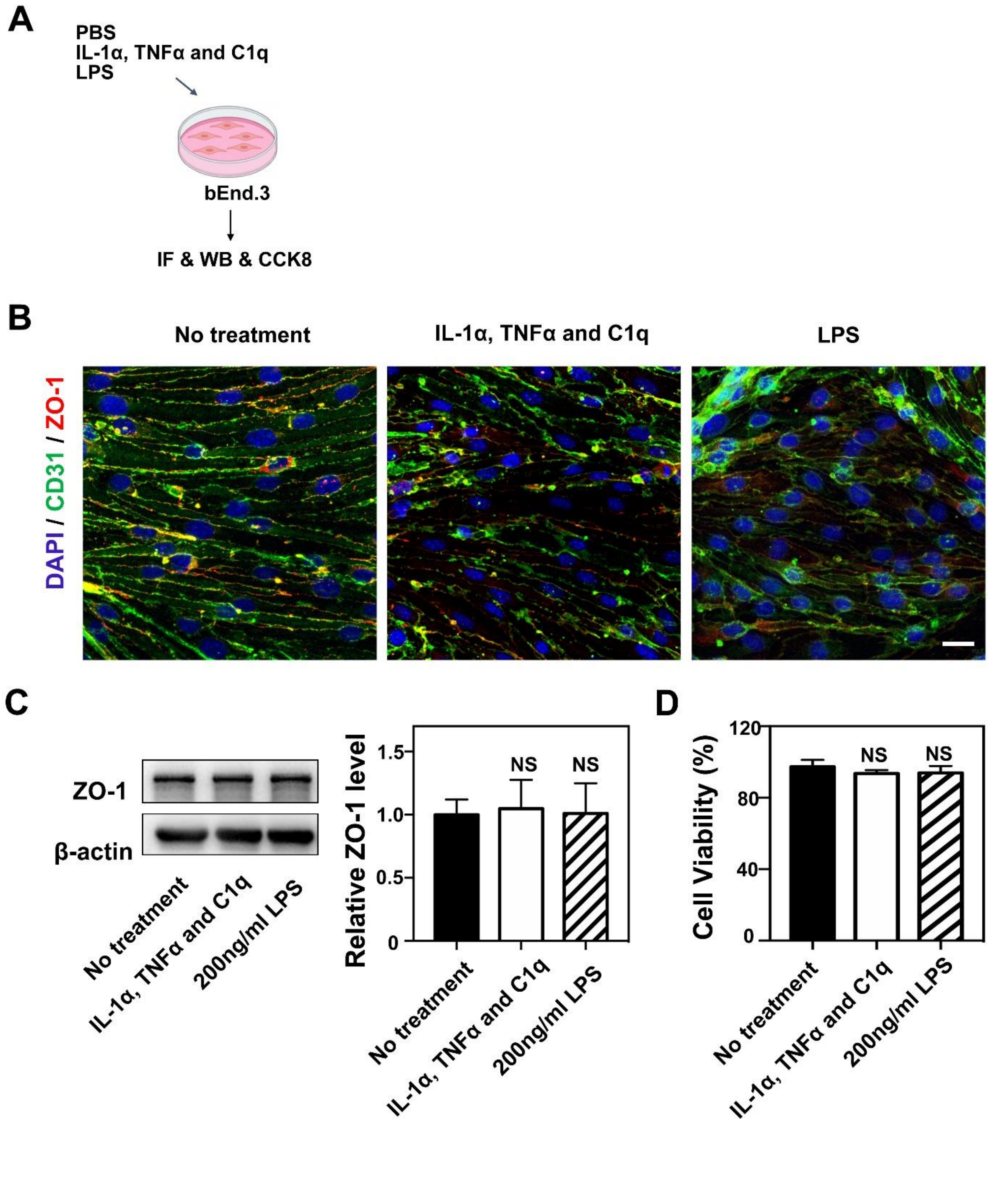

Supplementary Figure 3. IL-1α (3 ng/ml), TNFα (30 ng/ml) and C1q (400 ng/ml) or LPS (200 ng/ml) treatment did not affect the tight junction integrity and viability of bEnd.3 cells. A. bEnd.3 cells were treated with IL-1α+TNFα+C1q or LPS (200 ng/ml) for 24 hours, then immunocytochemistry and western blot were performed. **B.** Immunostaining of ZO-1/CD31 cells (ZO-1 in red color; CD31 in green color; DAPI in blue color). Scale bar=25 μm. **C.** Western blot of ZO-1 in bEnd.3 cells. Bar graph showed ZO-1 level in bEnd.3 cells. Data are mean ± SEM. n=3 per group. NS, *p*>0.05. **D.** CCK8 assay in bEnd.3 cells in no treatment group or treated with IL-1α+TNFα+C1q or 200 ng/ml LPS.

© 2021. Zhang Q et al. Published online at http://www.aginganddisease.org/EN/10.14336/AD.2021.1029

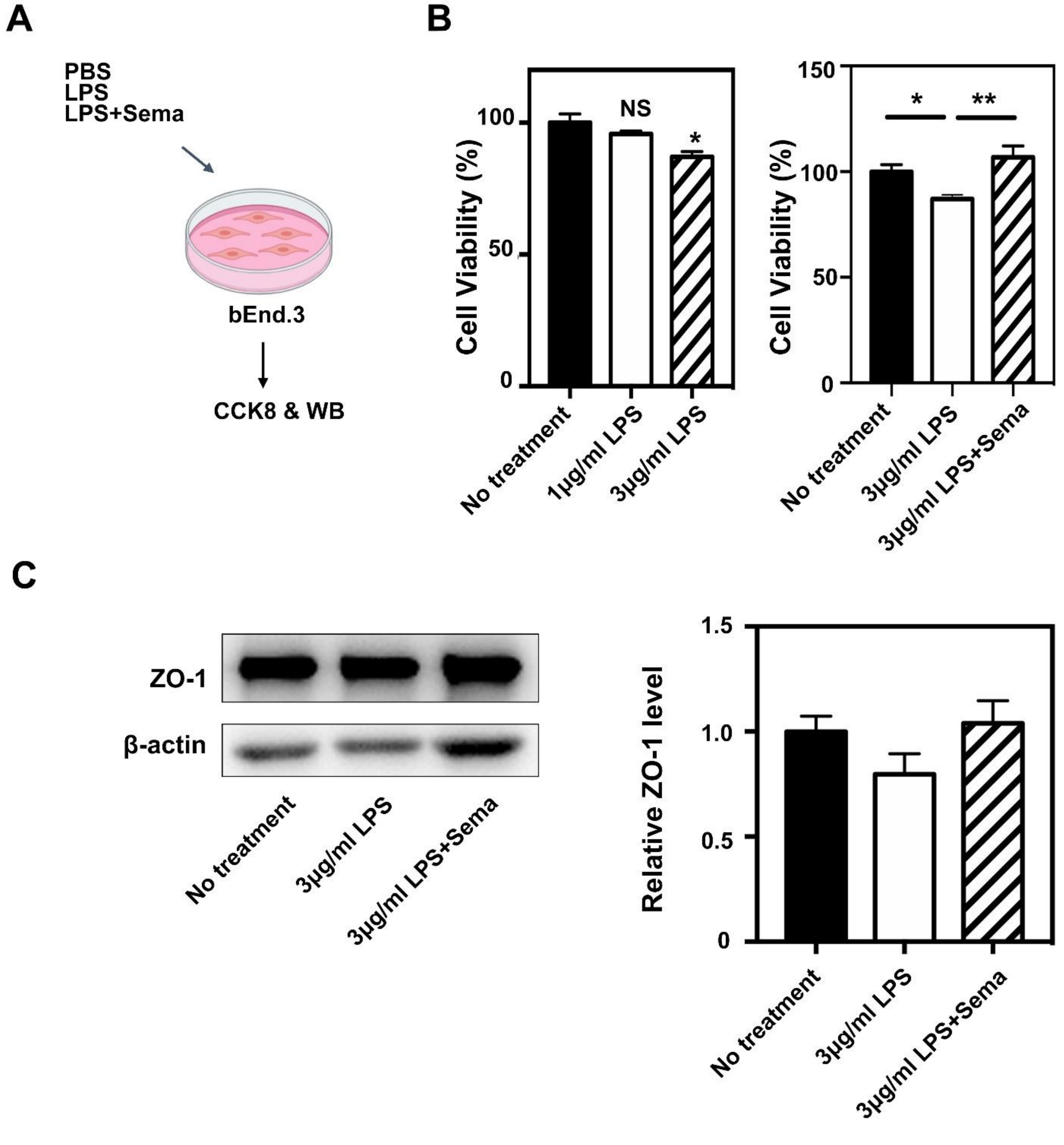

Supplementary Figure 4. Semaglutide treatment increased TJ integrity of bEnd.3 cells exposed to 3μg/ml LPS. A. bEnd.3 cells were treated with LPS or LPS+semaglutide for 24 hours, then CCK-8 and western blot were performed. **B.** CCK-8 assay showed the viability of bEnd.3 cells exposed to different concentration of LPS (1μg/ml and 3μg/ml). Data are mean ± SEM. n=8 per group. *, $p<0.05$; **, $p<0.01$. **C.** Western blot data showed the expression of ZO-1 in bEnd.3 cells treated with 3μg/ml LPS or 3μg/ml LPS+semaglutide. Data are mean ± SEM. n=3 per group.

© 2021. Zhang Q et al. Published online at http://www.aginganddisease.org/EN/10.14336/AD.2021.1029

SUPPLEMENTARY DATA

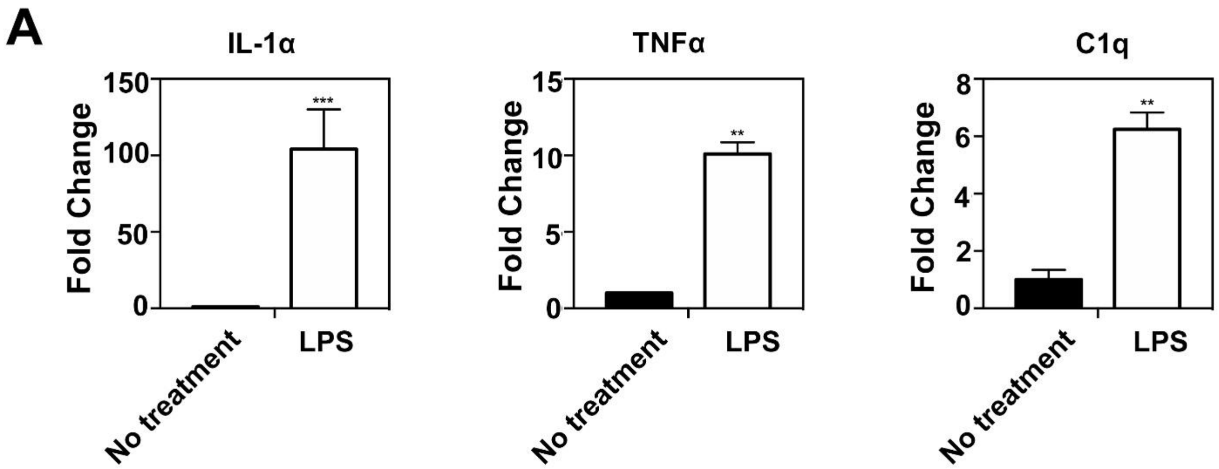

Supplementary Figure 5. LPS treatment increased the expression of IL-1α, TNFα and C1q in primary murine microglia. A. Bar graph showed the expression of IL-1α, TNFα and C1q in microglia after LPS treatment. Data are mean ± SEM. n=3 per group. **, *p*<0.01; ***, *p*<0.001.

© 2021. Zhang Q et al. Published online at http://www.aginganddisease.org/EN/10.14336/AD.2021.1029
